# Knowledge, Attitude and Practice regarding Breast Self-Examination among the Staff Nurses of Tertiary Care Hospital: An Observational Study

**DOI:** 10.31729/jnma.9192

**Published:** 2025-08-31

**Authors:** Rita Kumari Mahaseth, Rashika Upadhyaya, Prakash Chandra Panjiyar, Neha Jha, Nabina Singh, Rohan Rathod

**Affiliations:** 1 Nepal Medical College and Teaching Hospital, Attarkhel, Kathmandu, Nepal; 2 Helping Hands Community Hospital, Chabahil, Kathmandu, Nepal; 3 Nepal Army Institute of Health Sciences, Sanobharyang, Kathmandu, Nepal; 4 Kathmandu Medical College and Teaching Hospital, Sinamangal, Kathmandu, Nepal

**Keywords:** *breast self-examination*, *knowledge*, *attitude*, *practice*, *nurses*

## Abstract

**Introduction:**

Breast cancer is a leading public health concern for women in Nepal, making early detection critically important. Nurses serve as vital patient educators, and their own knowledge, attitude, and practice regarding breast self-examination are therefore essential. The study was aimed to find out the level of knowledge, attitude, and practice concerning breast self-examination among staff nurses at a tertiary care hospital.

**Methods:**

A descriptive cross-sectional study was conducted in a tertiary care hospital in Kathmandu, Nepal from June 1st to September 30th, 2025. Ethical clearance was obtained from the Institutional Review Committee (Reference number: NMC/IRC/01/24-25). A total of 180 staff nurses were included in the study using a total enumerative sampling method. Data were collected with a pretested, self-administered questionnaire based on previous literature. Data were entered and analyzed using Statistical Package for the Social Sciences version 23.0.

**Results:**

Among the 180 nurses, a good level of knowledge regarding breast selfexamination was found in 70 (38.89%) participants. A favorable attitude was seen in 74 (41.11%) nurses. Regarding practice, 133 (73.89%) participants reported performing the examination on a monthly basis.

**Conclusions:**

While a large proportion of participants reported performing BSE on a monthly basis, gaps in technical knowledge and variability in attitudes were identified. These findings highlight the coexistence of regular practice with variable knowledge and mixed perceptions among nurses.

## INTRODUCTION

Breast Self-Examination (BSE) is directly linked with improved survival rates for women diagnosed with breast cancer.^1^ This is especially important because breast cancer remains one of the leading causes of cancer-related deaths globally, and in developing countries like Nepal, late diagnosis due to limited public awareness is a common issue.^2^ Raising awareness of preventive measures like BSE is therefore crucial in reducing breast cancer morbidity and mortality across the nation.^3^

Although BSE is simple, non-invasive, and highly cost-effective, knowledge about the correct method and appropriate timing is often inadequate, even among students.^4^ A metaanalysis in Nepal highlights lack of sufficient knowledge about BSE, underscoring a significant gap.^5^ Since nurses are central to community health education, it is vital to assess their knowledge, attitude, and practice regarding BSE, as they are the primary individuals responsible for addressing this awareness gap.

Since nurses play a central role in providing health education to communities, it is vital to assess their knowledge, attitude, and practice regarding BSE, as they are the primary individuals responsible for addressing this awareness gap.

The purpose of this study was to determine the knowledge, attitude, and practice concerning breast self-examination among staff nurses working in a tertiary care hospital in Kathmandu, Nepal.

## METHODS

A descriptive cross-sectional study was conducted to determine the knowledge, attitude, and practice regarding breast selfexamination among staff nurses. The study was conducted in a tertiary care teaching hospital in Kathmandu, Nepal, over a period of four months, from June 1st, 2024, to September 30th, 2024. The study population comprised all staff nurses working in the clinical departments of the study site. A total enumerative sampling technique was used, wherein all eligible nurses who were present during the data collection period were included.

Ethical clearance was obtained from the Institutional Review Committee (Reference number: NMC/IRC/01/24-25). Written informed consent was secured from each participant before their enrolment in the study. The confidentiality and anonymity of all participants were maintained throughout the research process.

Inclusion criteria included all female staff nurses who had been working in any clinical department for at least six months and provided written informed consent. Exclusion criteria were nurses with a prior diagnosis or history of treatment for breast cancer, nurses who were pregnant during the study period, nurses with any cognitive impairment that would prevent informed consent, and male nurses.

Since the total number of staff nurses in the hospital (N=200) , a census of all available and eligible nurses was conducted. After excluding the 20 nurses involved in pre-testing the questionnaire, the final study population consisted of 180 nurses.

The primary variables were knowledge, attitude, and practice regarding BSE. Knowledge was defined as the correct responses to a set of questions about breast cancer risk factors, signs, and the steps, timing, and frequency of BSE. Attitude was defined as the nurses’ beliefs and perceptions towards the importance and effectiveness of BSE. Practice was defined as the regular and correct performance of BSE by the nurses themselves. The levels of knowledge, attitude, and practice were categorized based on a scoring system. For the knowledge section, each correct answer was awarded 1 point and each incorrect answer 0 points. For the attitude section, a 5-point Likert scale was used, with scores ranging from 1 to 5 after reverse scoring negative items from 5 to 1. The total scores for Knowledge, Attitude was; each section were converted into percentages (Percentage Score = (Participant’s Score / Maximum Possible Score) × 100). The final levels were determined using a Modified Bloom’s Cutoff which was based on the guidelines for KAP studies, where scores >80% were considered ‘Good,’ 60-80% were ‘Moderate,’ and <60% were ‘Poor.^6-10^

Data were collected using a structured, self-administered questionnaire. The instrument was developed following an extensive review of literature from similar Knowledge, Attitude, and Practice (KAP) studies conducted in the Nepalese context.^3^-^6^"^10^ The instrument was divided into four parts: sociodemographic characteristics, knowledge, attitude, and practice. The knowledge section comprised 8 items assessing key domains, including the appropriate timing and frequency of BSE (2 items), the correct technique for examination (5 items), and the recognition of signs of breast cancer (1 item). The attitude section included 13 statements measured on a 5-point Likert scale. The practice section consisted of 5 items assessing the frequency of BSE performance and health-seeking behaviors. To ensure content validity, the draft questionnaire was submitted to a panel of three subject matter experts, including a senior oncology nurse and a nursing faculty member, for review of its relevance, clarity, and completeness. A pretesting was then conducted on 20 staff nurses (10% of the target population), who were later excluded from the final analysis. Feedback from the pretesting was used to refine ambiguous wording and improve the flow of questions. Furthermore, the data from the pretesting were used to assess the internal consistency of the attitude scale, which yielded a Cronbach’s alpha of 0.78, indicating good reliability.

The collected data were entered and managed using Microsoft Excel 2016. Analysis was performed using the Statistical Package for the Social Sciences (SPSS) version 23.0. Frequency and percentages for binary data, and mean.

## RESULTS

A total of 180 nurses participated in the study. Specific knowledge regarding Breast Self-Examination (BSE) was evaluated. When asked about the most appropriate time for performing BSE, 110 (61.11%) participants selected ‘Between day 7 until day 10 after menstruation’. For menopausal and pregnant women, 122 (67.78%) participants chose ‘Same day each month’ as the appropriate time. The first step of BSE was identified as ‘Stand in front of mirror being shirtless and braless’ by 132 (73.34%) participants. The ‘Supine Position’ was identified as the appropriate position for palpation by 147 (81.67%) participants. ‘Use of the pads/pulps of three middle fingers’ was selected as the correct method for palpation by 55 (30.56%) participants. ‘Breast Lumps’ were identified as the earliest sign of breast cancer by 152 (84.44%) participants (Table 1).

In terms of BSE practice, 133 (73.89%) participants reported performing BSE monthly. Regarding advising others, 96 (53.33%) frequently gave advice to their parents or partner and 99 (55.00%) frequently gave advice to their friends and family. A total of 180 (100.00%) participants stated they would visit a health care center if a breast abnormality was noted (Table 2).

Participants’ attitudes towards BSE were recorded on a Likert scale. For the statement ‘All women should do BSE’, 129 (71.67%) participants strongly agreed and 47 (26.11%) agreed. In response to ‘Doing BSE is a waste of time’, 80 (44.44%) disagreed and 98 (54.44%) strongly disagreed. When asked if they would prefer treatment from a traditional healer for a lump, 85 (47.22%) disagreed and 86 (47.78%) strongly disagreed. Regarding the statement ‘I am interested in doing BSE’, 72 (40.00%) strongly agreed and 70 (38.89%) agreed (Table 3).

The overall assessment of knowledge revealed that 70 (38.89%) participants had a good knowledge level, 80 (44.44%) had a moderate knowledge level, and 30 (16.67%) had a poor knowledge level (Table 4). The assessment of attitude showed that 74 (41.11%) participants had a favorable attitude, 40 (22.22%) had a neutral attitude, and 66 (36.67%) had an unfavorable attitude (Table 4).

**Table 1:** Knowledge regarding Breast Self Examination (n = 180).

Items	n (%)
Most appropriate time for performing BSE	
Between day 7 until day 10 after menstruation	110(61.11)
Any time of the month	8(4.44)
Day 4 after menstruation	62(34.45)
Appropriate time for performing BSE in menopausal and pregnant women	
Not Necessary	1 (6.11)
Same day each month	122(67.78)
Twice in a month	31(17.22)
Don't Know	16(8.89)
First step of Breast self examination	
Visual examination of breast	40(22.22)
Stand in front of mirror being shirtless and braless	132(73.34)
Palpation of breast	8(4.44)
Appropriate position for palpation in BSE	
Lateral Position	27(15.00)
Supine Position	147(81.67)
Don't Know	6(3.33)
Proper technique during palpation in BSE	
Hand should be raised up alternatively above the head	146(81.11)
Examine both breasts in mirror with hands on hip	18(10.00)
Hand should be raised up together above the head	16(8.89)
Correct method while doing palpation in BSE	
Use of the tip of three middle fingers	123(68.33)
Whole fingers	2(1.11)
Use of the pads/pulps of three middle fingers	55(30.56)
Correct technique for breast palpation	
Vertical stripe and circular technique	50(27.78)
Horizontal stripe and circular technique	22(12.22)
Only circular technique	105(58.33)
Don't Know	3(1.67)
Earliest sign of breast cancer	
Breast Lumps	152(84.44)
Unusual Discharge	16(8.89)
Skin irritation around breast	12(6.67)

**Table 2:** Practice regarding Breast Self Examination (n= 180).

Characteristics	n (%)
Frequency of performing BSE	
Never	11(6.11)
Monthly	133(73.89)
Bi-annually	25(13.89)
Quarterly	11(6.11)
Frequency of advice from parents or partner for performing BSE	
Never	40(22.22)
Seldom	18(10.00)
Frequent	96(53.33)
Always	26(14.45)
Frequency of advice to friends and family for performing BSE	
Never	13(7.22)
Seldom	19(10.56)
Frequent	99(55.00)
Always	49(27.22)
Frequency of discussion about importance of BSE with friends and family	
Never	10(5.56)
Seldom	38(21.11)
Frequent	98(54.44)
Always	34(18.89)
Practice if breast abnormality is noted	
Visit health care center	180(100.00)

**Table 4:** Level of Knowledge and Attitude Regarding Breast Self-Examination using Modified Bloom Cut-off (n=180).

Variable	Category	n(%)
Knowledge Level		
	Good	70(38.89)
	Moderate	80(44.44)
	Poor	30(16.67)
Attitude Level		
	Favorable	74(41.11)
	Neutral	40(22.22)
	Unfavorable	66(36.67)

**Table 3:** Attitude regarding Breast Self Examination (n= 180).

Characteristics	Strongly Agree	Agree	Neutral	Disagree	Strongly Disagree
Doing BSE makes me funny.	3(1.67)	6(3.33)	20(11.11)	84(46.67)	67(37.22)
BSE will be embarrassing to me.	2(1.11)	6(3.33)	6(3.33)	89(49.44)	77(42.78)
Doing BSE is a waste of time.	-	1(0.56)	1(0.56)	80(44.44)	98(54.44)
Doing BSE makes me feel unpleasant.	-	5(2.78)	16(8.89)	89(49.44)	70(38.89)
If there is a lump, I prefer to get treatment from the traditional healer.	-	-	9(5.00)	85(47.22)	86(47.78)
Cannot do BSE once in a month as it feels uncomfortable.	3(1.67)	12(6.67)	13(7.22)	93(51.67)	59(32.78)
All women should do BSE.	129(71.67)	47(26.11)	4(2.22)	-	-
I really care about my breast.	122(67.78)	53(29.44)	5(2.78)	-	-
I am not afraid to think about breast cancer.	18(10.00)	27(15.00)	40(22.22)	59(32.78)	36(20.00)
I avoid BSE because I worry about breast cancer.	8(4.44)	5(2.78)	15(8.33)	90(50.00)	62(34.44)
I am interested in doing BSE.	72(40.00)	70(38.89)	34(18.89%	4(2.22)	-
I always search for the information regarding BSE from internet, magazine, newspaper.	44(24.44)	84(46.67)	51(28.33)	-	1(0.56)
I discuss with my friends and family about BSE.	50(27.81	83(46.11)	47(26.11)	-	-

## DISCUSSION

This study revealed a notable finding in the knowledge, attitude, and practice regarding Breast Self-Examination (BSE) among staff nurses in a tertiary care hospital in Nepal. While the rate of monthly BSE practice was high, many lacked a consistently favorable attitude. The level of knowledge in our study, with 38.89% of nurses having ‘good’ knowledge, presents a differnt picture when compared to other research. This finding is higher than the 8.7% with good knowledge among health science students in Ethiopia, and similar finding with studies of the general Nepalese population in Butwal, where over half the women had poor knowledge.^11,12^ However, it is less than 100% awareness of BSE reported among nurses in another Nepalese study in Pokhara.^10^ This variability may suggest that while a nursing background provides a foundational understanding better than general public or students, there may be difference across different institutions and regions.

A significant finding of this study was the variable attitude among participants; while 41.11% had a favorable attitude, 58.89% had either neutral or unfavorable views. The past literature has shown that formal BSE can lead to “psychological harms,” such as anxiety from false-positive findings and an increase in unnecessary biopsies, which may contribute to a unfavourable attitude even among professionals who know the procedure.^13,14^ This finding aligns with research that has identified misconceptions and fear as significant barriers to to engagement with breast health screening.^8^

The reported monthly practice of BSE by nurses in this study (73.89%) was substantially higher than that of the general population.^7,12^ However, this finding must be compared to the current medical landscape, near BSE’s efficacy. Two RCT trials and a Cochrane review concluded that routine, formal BSE does not reduce breast cancer mortality and increases the rate of benign biopsies.^14^ Consequently, major bodies like the United States Preventive Service Task Force (USPSTF) do not recommend teaching formal BSE.^13^ However, the clinical importance of early detection remains undisputed, as delayed presentation of breast cancer symptoms is directly linked to poorer survival outcomes.^15^ Consequently, the high practice rate among nurses may be more appropriately interpreted as a form of effective ‘breast awareness’.

The study has limitations. Its single-center design restricts the generalization of the findings to the broader nursing population in Nepal. Furthermore, the cross-sectional nature of the study precludes any inference of causality, and the reliance on selfreported data for practice is a potential source of social desirability bias. A limitation of this study is the use of the Modified Bloom’s Cutoff. The percentage cutoffs in this method are traditional and somewhat arbitrary, and they are not proven to be accurate for this specific study. This method also turns detailed scores into broad groups, which can hide small differences. Even so, it was used because it is common in similar studies, makes comparison easier, and clearly summarizes the results.

The results of this study are important. The gaps in knowledge, along with ongoing debate about formal BSE, suggest that in low-resource settings, training should move away from strict monthly BSE rules and instead focus on a broader idea of “breast awareness.” This means teaching nurses and through them, their patients to notice any changes in their breasts and seek medical help quickly, without unnecessary worry. Since educational programs have already shown good results, this type of training could work well.^16^ Future research should look at creating and testing breast awareness programs suited to Nepal. Qualitative studies could also help understand why some nurses had negative attitudes.

## CONCLUSIONS

While a large proportion of participants reported performing BSE on a monthly basis, gaps in technical knowledge and variability in attitudes were identified. These findings highlight the coexistence of regular practice with variable knowledge and mixed perceptions among nurses.
